# No Association between the Plasmodium vivax *crt-o* MS334 or In9*pvcrt* Polymorphisms and Chloroquine Failure in a Pre-Elimination Clinical Cohort from Malaysia with a Large Clonal Expansion

**DOI:** 10.1128/aac.01610-22

**Published:** 2023-06-14

**Authors:** Angela Rumaseb, Roberto R. Moraes Barros, Juliana M. Sá, Jonathan J. Juliano, Timothy William, Kamil A. Braima, Bridget E. Barber, Nicholas M. Anstey, Ric N. Price, Matthew J. Grigg, Jutta Marfurt, Sarah Auburn

**Affiliations:** a Global and Tropical Health Division, Menzies School of Health Research and Charles Darwin University, Darwin, Northern Territory, Australia; b Laboratory of Malaria and Vector Research, National Institute of Allergy and Infectious Diseases, National Institutes of Health, Bethesda, Maryland, USA; c Division of Infectious Diseases, School of Medicine, University of North Carolina at Chapel Hill, Chapel Hill, North Carolina, USA; d Clinical Research Centre, Queen Elizabeth Hospital, Sabah, Malaysia; e Infectious Diseases Society Sabah-Menzies School of Health Research Clinical Research Unit, Kota Kinabalu, Sabah, Malaysia; f QIMR Berghofer Medical Research Institute, Brisbane, Australia; g Centre for Tropical Medicine and Global Health, Nuffield Department of Medicine, University of Oxford, Oxford, United Kingdom; h Mahidol-Oxford Tropical Medicine Research Unit, Mahidol University, Bangkok, Thailand; i College of Medicine and Public Health, Flinders University, Darwin, Northern Territory, Australia; j Department of Microbiology, Immunology and Parasitology, Escola Paulista de Medicina, Universidade Federal de São Paulo, São Paulo, Brazil

**Keywords:** *Plasmodium vivax*, malaria, chloroquine, antimalarial, drug resistance, molecular marker, *pvcrt-o*, Malaysia

## Abstract

Increasing reports of resistance to a frontline malaria blood-stage treatment, chloroquine (CQ), raises concerns for the elimination of Plasmodium vivax. The absence of an effective molecular marker of CQ resistance in P. vivax greatly constrains surveillance of this emerging threat. A recent genetic cross between CQ sensitive (CQS) and CQ resistant (CQR) NIH-1993 strains of P. vivax linked a moderate CQR phenotype with two candidate markers in P. vivax CQ resistance transporter gene (*pvcrt-o*): MS334 and In9*pvcrt*. Longer TGAAGH motif lengths at MS334 were associated with CQ resistance, as were shorter motifs at the In9*pvcrt* locus. In this study, high-grade CQR clinical isolates of P. vivax from a low endemic setting in Malaysia were used to investigate the association between the MS334 and In9*pvcrt* variants and treatment efficacy. Among a total of 49 independent monoclonal P. vivax isolates assessed, high-quality MS334 and In9*pvcrt* sequences could be derived from 30 (61%) and 23 (47%), respectively. Five MS334 and six In9*pvcrt* alleles were observed, with allele frequencies ranging from 2 to 76% and 3 to 71%, respectively. None of the clinical isolates had the same variant as the NIH-1993 CQR strain, and none of the variants were associated with CQ treatment failure (all *P > *0.05). Multi-locus genotypes (MLGs) at 9 neutral microsatellites revealed a predominant P. vivax strain (MLG6) accounting for 52% of Day 0 infections. The MLG6 strain comprised equal proportions of CQS and CQR infections. Our study reveals complexity in the genetic basis of CQ resistance in the Malaysian P. vivax pre-elimination setting and suggests that the proposed *pvcrt-o* MS334 and In9*pvcrt* markers are not reliable markers of CQ treatment efficacy in this setting. Further studies are needed in other endemic settings, applying hypothesis-free genome-wide approaches, and functional approaches to understand the biological impact of the TGAAGH repeats linked to CQ response in a cross are warranted to comprehend and track CQR P. vivax.

## INTRODUCTION

Plasmodium vivax malaria remains an important public health burden affecting the poorest and most vulnerable communities of more than 49 endemic countries (1, 2). Chloroquine (CQ) is still the most widely used antimalarial drug to treat P. vivax but increasing reports of CQ resistance highlight the importance of surveillance of its clinical efficacy (3). Although surveillance of clinical efficacy is the gold standard for the detection of CQ resistance, challenges in discriminating infections deriving from blood-stage failures (recrudescence), new mosquito inoculations (reinfections), and reactivation of dormant liver stages (relapses) confounds robust clinical estimates of CQ resistance (4). Molecular surveillance presents a more accurate and cost-effective strategy to identify drug resistant infections (5). However, the genetic basis of CQ resistance in P. vivax remains unclear.

Several studies have investigated Single Nucleotide Polymorphisms (SNPs) in orthologues of genes involved in CQ resistance in Plasmodium falciparum, namely, *pvcrt-o*, and *pvmdr1*, but none of these markers have been confirmed to be associated with clinical outcomes following CQ treatment of P. vivax (6–9). Other studies have reported an association between *pvcrt-o* expression levels and P. vivax CQ resistance (10–13). However, no evidence of association was observed between *pvcrt-o* expression and *ex vivo* drug susceptibility phenotypes using clinical isolates from Papua Indonesia (14), an area where high rates of clinical failure have been documented following CQ treatment (15).

More recently, a genetic cross between P. vivax subpopulations differing in susceptibility to CQ in nonhuman primates identified 2 new, non-coding *pvcrt-o* markers associated with *in vivo* CQ sensitivity: a repeat length polymorphism (MS334) and an intron 9 polymorphism (In9*pvcrt*) (12). The MS334 locus is located approximately 0.6 Kb upstream of the start codon of *pvcrt-o;* CQ sensitive (CQS) progeny contained 10 TGAAGH motifs, while CQ resistant (CQR) progeny carried a longer fragment of 15 TGAAGH motifs. At the In9*pvcrt* locus, 17 TGAAGH motifs were present in CQS progeny, whereas a shorter fragment comprising 14 TGAAGH motifs were present in CQR progeny.

A prospective clinical trial conducted in Sabah, Malaysia in 2016 observed that more than 60% of P. vivax-infected patients treated with CQ had recurrent parasitemia within 28 days of treatment, indicative of high-grade CQ resistance (3, 4, 16). Microsatellite-based genotyping of 17 Day 0 and recurrent infection pairs confirmed that they were genetically homologous (16). Subsequent analysis of the microsatellite genotyping data also revealed evidence of clonal expansions (17). However, it is unclear whether these clonal expansions were involved in CQ resistance. The aim of the current study was to further explore the underlying parasite genetic diversity and the potential role of *pvcrt-o* MS334 and In9*pvcrt* polymorphism in determining patients’ clinical outcomes following CQ treatment in Malaysia.

## RESULTS

### Summary of patient samples, sequencing and genotyping data.

**FIG 1:**
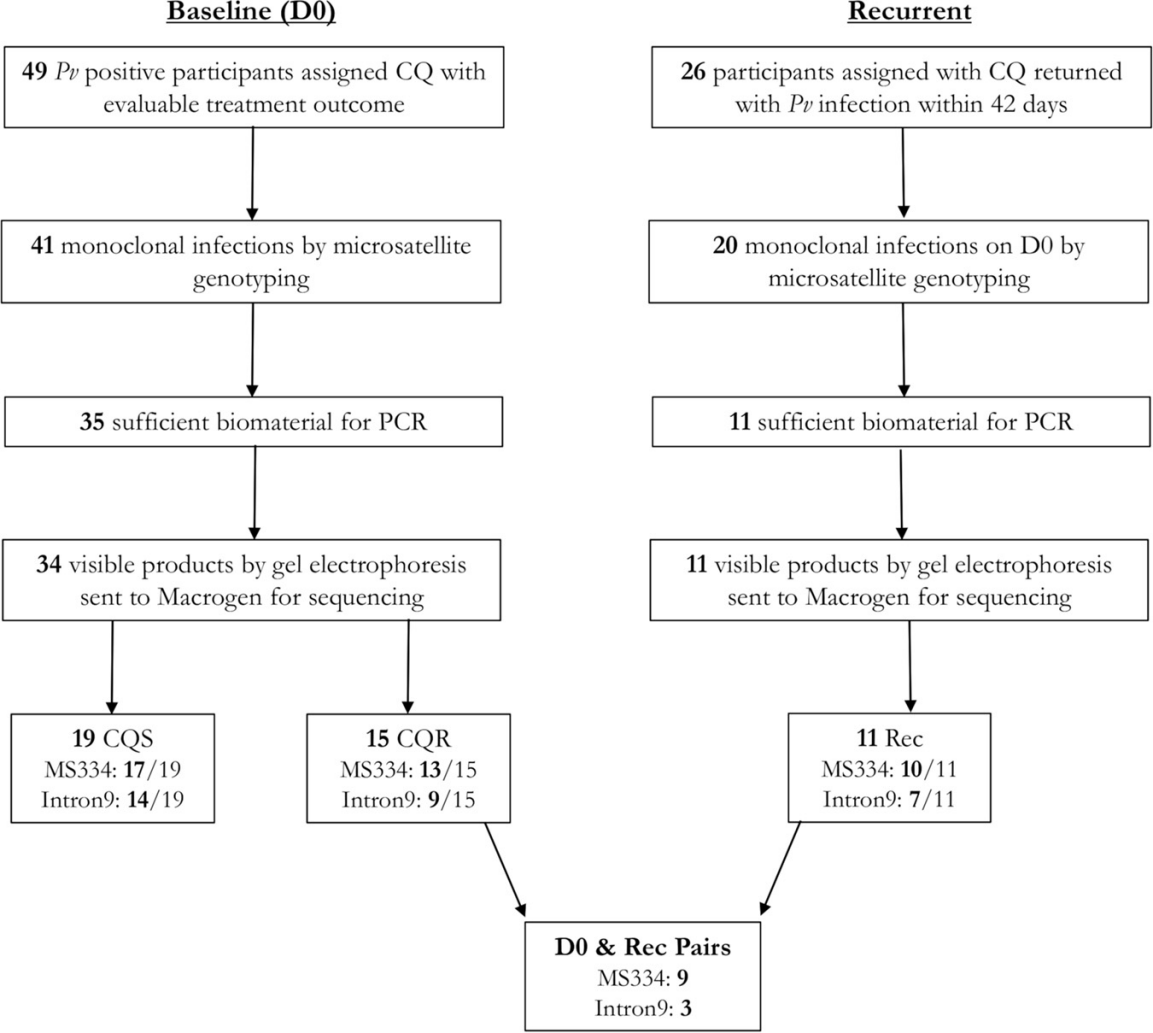
Flowchart outlining patient sample exclusion. *Pv*, Plasmodium vivax*;* CQ, Chloroquine; PCR, polymerase chain reaction; CQS, chloroquine sensitive; CQR, chloroquine resistant; Rec, recurrent infection time point.

The filtering process for all samples used in this study is presented in Fig. 1. Among 49 clinical samples collected at baseline from patients treated with CQ, high-quality sequences could be derived from independent monoclonal P. vivax infections for MS334 in 30 (61%) isolates and In9*pvcrt* in 23 (47%) isolates. An additional 9 high-quality sequences at MS334 and 3 at In9*pvcrt* were also derived in paired isolates at baseline and day of recurrence. Details of the clinical characteristics of the patients and assay results of the isolates are presented in File S1. There were no significant differences in age, gender, and baseline parasitemia between the *pvcrt-o* study set and all patients treated with CQ in the trial (Table 1).

**TABLE 1:** Demographic data of CQ-assigned participants in clinical trial and *pvcrt-o* study set

	Age^*a*^ *N* (%)			Males *N* (%)	Asexual parasitemia^*b*^ median (range)
Measure	<5	5 to 15	>15		
Baseline^*c*^	1/49 (2.04)	23/49 (46.94)	25/49 (51.02)	33/49 (67.35)	3,131 (240–42,521)
Study^*d*^	0/34 (0)	17/34 (50)	17/34 (50)	20/34 (58.82)	2,933 (408–42,521)
Test statistic (*p-value*)	X^2^ = 2.218e-31 (*p *= 1)	X^2^ = 0.003 (*p *= 0.959)	X^2^ = 0 (*p *= 1)	X^2^ = 0.316 (*p *= 0.574)	W = 820 (*p *= 0.908)

a Age in years.

b Parasites/μL.

c Participants assigned CQ on baseline.

d Participants included in the *pvcrt-o* study set.

Sequencing data on the successfully sequenced and aligned MS334 and In9*pvcrt* amplicons are deposited in GenBank under accession numbers OP807877 - OP807946, respectively. The sequencing pass rates among the baseline and recurrent infections were 89% (40/45) for MS334 and 67% (30/45) for In9*pvcrt*.

All but 1 sample (97.5% [39/40]) with successfully sequenced and aligned MS334 or In9*pvcrt* amplicons had genotype calls at all 9 microsatellite markers. The microsatellite-based multi-locus genotypes (MLGs) are presented in File S1.

### Summary of baseline genetic diversity in Malaysia using microsatellite genotyping.

**FIG 2:**
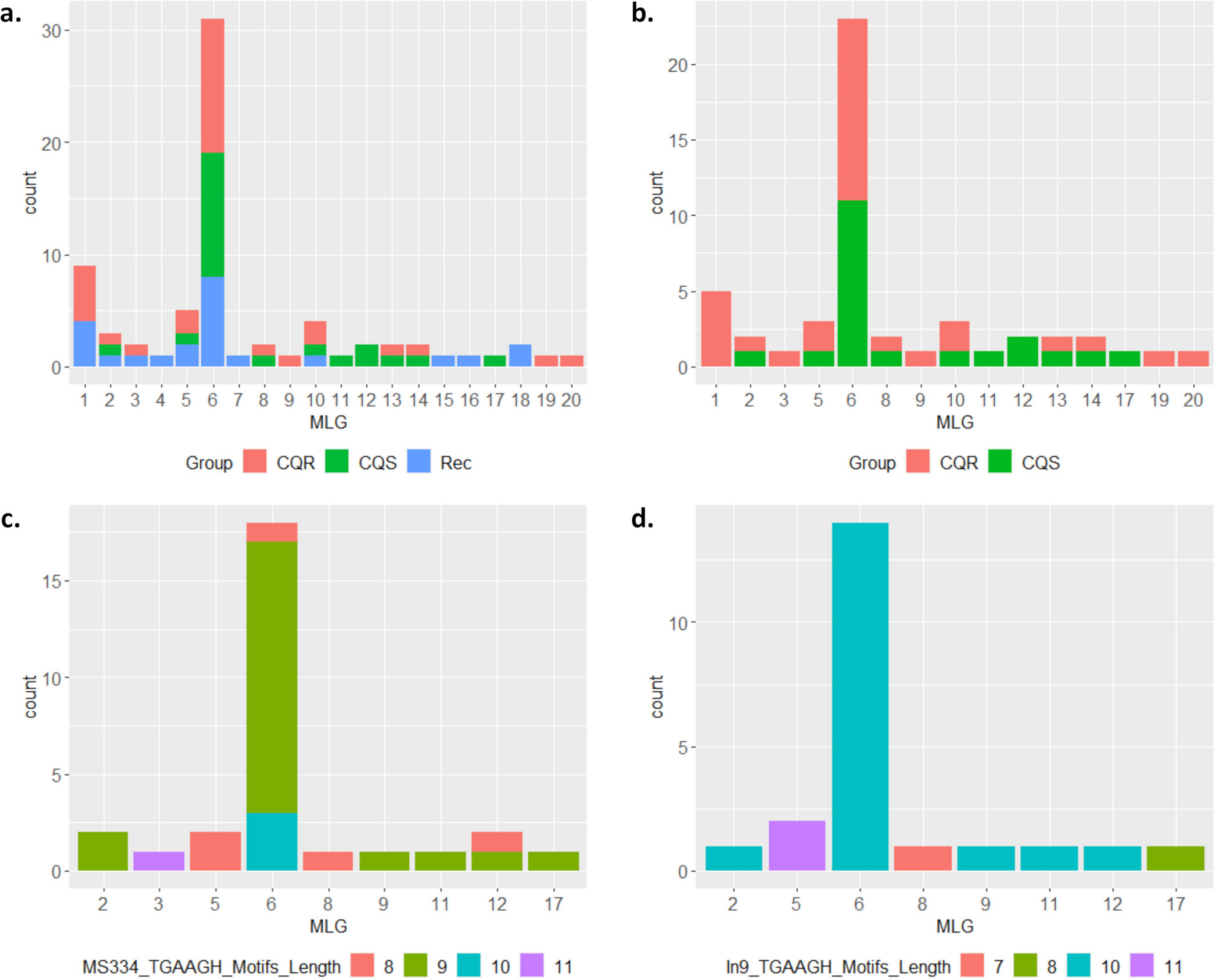
Bar charts illustrating the distributions of the microsatellite-based multi-locus genotypes (MLGs) by CQ outcome and MS334 and In9*pvcrt* variant. (a) MLGs observed in all (Day 0 and recurrent) P. vivax isolates with breakdown by CQ treatment outcome. (b) MLGs observed in the baseline (Day 0) P. vivax isolates, with breakdown by CQ treatment outcome. (c) MLGs observed in the baseline (Day 0) P. vivax isolates, with breakdown by TGAAGH motifs at *pvcrt-o* MS334. (d) MLGs observed in the baseline (Day 0) P. vivax isolates, with breakdown by TGAAGH motifs at *pvcrt-o* Intron9. MLG, microsatellite-based multi-locus genotype; CQ, Chloroquine; CQS, chloroquine sensitive (day 0 infections); CQR, chloroquine resistant (day 0 infections); Rec, recurrent infection time point; *pvcrt-o*, Plasmodium vivax chloroquine resistance transporter orthologue.

A summary of the microsatellite-based multi-locus genotypes (MLGs) observed in Malaysian P. vivax isolates is presented in Fig. 2 and listed in File S2. Across the 39 Day 0 and recurrent paired isolates, a total of 20 MLGs were observed (15 MLGs among the Day 0 infections), demonstrating moderate diversity within the population (Fig. 2a). However, 79% of infections had a single MLG (MLG6), highlighting a predominating strain. On restricting analyses to the baseline (Day 0) infections, similar proportions were observed, with 52% of infections having an MLG6 genetic backbone (Fig. 2b). Exploration of CQ treatment outcome by MLG revealed no evidence of MLG6 as a marker of CQR, with a near equal distribution of CQS and CQR infections (Fig. 2a and b).

### Summary of MS334 and In9*pvcrt* variants observed in Malaysia.

A summary of the MS334 and In9*pvcrt* genotype calls is provided in Table 2. Five MS334 and six In9*pvcrt* insertion and deletion (indel) variants were identified among the Day 0 and recurrent Malaysian isolates, with allele frequencies ranging from 2% (1/41) to 76% (31/41) and 3% (1/31) to 71% (22/31), respectively. Representative sequences of the 5 MS334 and 6 In9*pvcrt* indels observed in Malaysia, as well as the NIH-1993-R and NIH-1993-S strains, aligned against the Sal-1 reference strain, are provided in Files S3 and 4.

**TABLE 2:** Summary of insertion and deletion variants observed in Malaysia

Candidate marker	Variant no.	Indel polymorphism(s)	Positions^*a*^	CQS	CQR	X^2^^*b*^ (*p-value*)	Rec^*c*^
MS334	1	None	NA	1/17	0/13	6.924e-33^*d*^ (*p *= 1)	0/10
	2	16bp insertion	−52	14/17	10/13	3.346e-32 (*p* = 1)	7/10
	3	16bp insertion 16 bp deletion	−52 +43	1/17	2/13	0.060^*d*^ (*p *= 0.806)	1/10
	4	16bp insertion 16 bp insertion^i^	−52 +59	1/17	0/13	6.924e-33^*d*^ (*p *= 1)	0/10
	5	32bp insertion 16 bp insertion^i^	−52 +59	0/17	1/13	0.019^*d*^ (*p *= 0.891)	2/10
In9*pvcrt*	1	None	NA	0/14	0/9	NA	1/7
	2	41bp deletion	+3,121	1/14	0/9	1.584e-31^*d*^ (*p *= 1)	0/7
	3	120bp deletion^i^	+3,135	1/14	1/9	1.259e-32^*d*^ (*p *= 1)	2/7
	4	140bp deletion^i^	+3,135	11/14	7/9	5.980e-31 (*p *= 1)	4/7
	5	220bp deletion^*e*^ 20 bp insertion	+3,095 +3,289	0/14	1/9	0.052^*d*^ (*p *= 0.820)	0/7
	6	180bp deletion	+3,095	1/14	0/9	1.584e-31^*d*^ (*p *= 1)	0/7

a Number of base pairs upstream (−) or downstream (+) from the start of *pvcrt-o* in Sal-1 strain (Pv_Sal1_chr01:330,260).

b Chi-squared test of CQS vs CQR baseline (i.e., not including the recurrent time point) isolates.

c Rec, recurrent infection time point. See Files S3 and 4 for sequence details for each variant.

d Note the statistical constraints of the limited number of samples representing the given variant.

e Part of polymorphisms observed in NIH-1993-R mentioned by (12); CQS, chloroquine sensitive; CQR, chloroquine resistant.

At the MS334 locus, longer variable number of TGAAGH motifs were associated with CQR in the NIH-1993-R × S strains (12). The NIH-1993-R type (inferred CQR type) MS334 allele comprising the 15 TGAAGH motifs was not observed in Malaysia, and all variants at this locus exhibited shorter motif lengths, ranging from 8 to 11 motifs. Most clinical isolates also contained motifs shorter than the NIH-1993-S (10 motifs) type.

**FIG 3:**
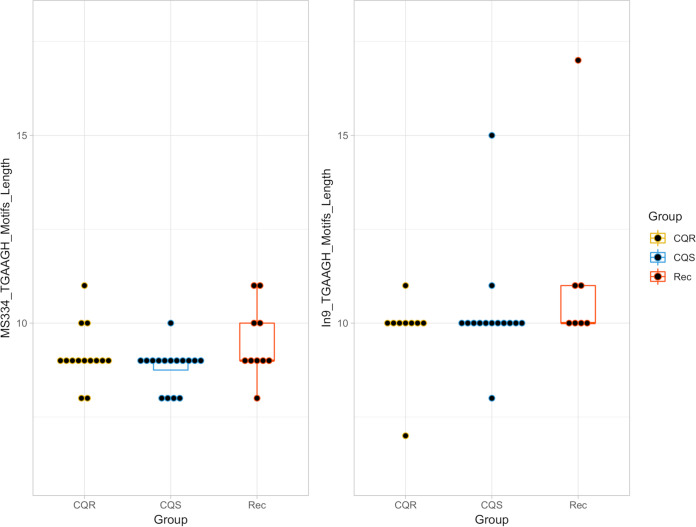
Dot-boxplots of numbers of TGAAGH motif lengths observed in Malaysian isolates. CQS, chloroquine sensitive (day 0 infections); CQR, chloroquine resistant (day 0 infections); Rec, recurrent infection time point.

At the In9*pvcrt* locus, shorter variable numbers of TGAAGH motifs were associated with CQR in the NIH-1993 strains (12). The NIH-1993-R type In9*pvcrt* allele comprising 14 TGAAGH motifs was not observed in the clinical isolates from Malaysia, but motif lengths ranging from 7 to 17 were observed. Most infections contained 10 TGAAGH motifs (22/31; 71%), which is shorter than both the NIH-1993-R (14 motifs) and NIH-1993-S (17 motifs) types. The distribution of the different TGAAGH (TGAAGC, TGAAGA, and TGAAGT) motif lengths observed in clinical CQS, CQR, and recurrent isolates are summarized in Fig. 3.

The distribution in the variable number of TGAAGH motifs for MS334 and In9*pvcrt* was explored by MLG to assess the independence in transmission of the alleles at these candidates relative to the MLG ‘backbone’ (Fig. 2c and d, respectively). Three motif length variants were observed among the MLG6 strains, suggesting modest (albeit low-level) independence between the MS334 and MLG markers. The most common MS334 variant among the MLG6 strains was a 9 TGAAGH motif accounting for 67% of the infections in this group, but this motif length was also observed in 5 other MLGs. All MLG6 infections had the same motif length at the In9*pvcrt* marker (10 TGAAGH motifs), but this variant was also observed in 4 other MLGs.

### Assessment of MS334 and In9*pvcrt* association with CQ treatment outcome.

There was no statistically significant difference in the proportion of any of the five MS334 or six In9*pvcrt* alleles (all *P > *0.05) between CQS and CQR clinical isolates (Table 2), although comparison was constrained by sample size at all variants other than MS334 variant 2 and *In9pvcrt* variant 4. None of the clinical isolates carried the exact motifs of the NIH-1993-R strains at the MS334 and In9*pvcrt* loci, the Malaysian MS334 variant types 4 and 5, and In9*pvcrt* variant types 3, 4 and 5 possessed fragments of the NIH-1993-R indels (Files S3 and 4). NIH-1993 is closely related to Salvador-I, a P. vivax isolate adapted to nonhuman primates prior to CQR spread and from a region where no stable or high CQR vivax malaria has been reported. There was no significant difference in the proportion of the combined MS334 variants 4 and 5 (χ^2^ = 4.016e-31, *P = *1) or combined In9*pvcrt* variants 3, 4, and 5 (χ^2^ = 1.584e-31, *P = *1) between the CQS and CQR isolates.

Further assessment of the MS334 and In9*pvcrt* variants was undertaken in paired isolates at baseline and the day of recurrent parasitemia. Seven of the 9 pairs successfully sequenced at MS334, and all 3 pairs sequenced at In9*pvcrt* exhibited the same alleles at both time points (Table 3). In the 2 pairs exhibiting allelic differences at MS334 (MV019 and TK026 pairs), the changes in variants pre and post treatment were not consistent. One pair possessed a longer allele at baseline, and the other pair possessed a longer allele at recurrence. Analysis of microsatellite-based genotyping data in 2 paired isolates revealed evidence of polyclonal infection at recurrence but not at baseline (Table 4).

**TABLE 3:** Summary of NIH-1993-R type insertion and deletion polymorphisms observed in paired Malaysian isolates^*a*^

		Insertion at 330,319^*b*^ (MS334)		Deletion at 333,355^*b*^ (*In9pvcrt*)	
Pair	Sample	Day 0	Day rec	Day 0	Day rec
1	MK079	-	✓	-	✗
2	MV010	✗	-	-	-
**3**	**MV019**	**✓**	**✗**	-	✓
4	MV021	✗	✗	✓	✓
5	MV033	✗	✗	-	✓
**6**	**TK026**	**✗**	**✓**	✓	-
7	TV029	✗	✗	-	✓
8	TV038	✗	✗	✓	-
9	TV040	✗	✗	✓	✓
10	TV048	✗	✗	✓	✓
11	TV051	✗	✗	-	-

a Infections with day 0 versus recurrence differences are highlighted in bold.

b Positions at chromosome 1 of Sal-1 reference strain; Rec, recurrent infection time point; (-) not amplified; (✓) insertion/deletion is present; (✗) insertion/deletion is not present.

**TABLE 4:** Comparison of microsatellite genotyping data and *pvcrt-o* MS334 and In9*pvcrt* markers results in paired Malaysian isolates^*a*^

Pair	Sample	MS12	pv3.27	msp1f3	MS10	MS5	MS1	MS8	MS16	MS20	MLG^*b*^	Indel mutation similarities	
												MS334	*In9pvcrt*
1	MK079	209	280	258	195	194	237	281	385	201	1	-	-
1	MK2079	209	280	258	195	194	237	281	385	201	1		
2	MV010	206	280	261	180	182	237	263	352	201	2	-	-
2	MV1010	206	280	261	180	182	237	263	352	201	2		
**3**	**MV019**	**209**	**280**	**258**	**195**	**182**	**237**	**281**	**385**	**219**	**3**	✗	-
**3**	**MV1019**	**209**	**280**	**258**	**195/153**	**182**	**237**	**281**	**385**	**219**	**3/4**		
4	MV021	209	280	348	195	164	228	281	463	201	5	✓	✓
4	MV1021	209	280	348	195	164	228	281	463	201	5		
5	MV033	209	280	255	195	173	237	281	418	201	6	✓	-
5	MV1033	209	280	255	195	173	237	281	418	201	6		
**6**	**TK026**	**209**	**280**	**255**	**195**	**173**	**237**	**281**	**418**	**201**	**6**	✗	-
**6**	**TK1026**	**209**	**280**	**255**	**195**	**173/182**	**237**	**281**	**418**	**201**	**6/7**		
7	TV029	209	280	255	195	173	237	281	418	201	6	✓	-
7	TV1029	209	280	255	195	173	237	281	418	201	6		
8	TV038	209	280	255	195	173	237	281	418	201	6	✓	-
8	TV1038	209	280	255	195	173	237	281	418	201	6		
9	TV040	209	280	255	195	173	237	281	418	201	6	✓	✓
9	TV1040	209	280	255	195	173	237	281	418	201	6		
10	TV048	209	280	255	195	173	237	281	418	201	6	✓	✓
10	TV1048	209	280	255	195	173	237	281	418	201	6		
11	TV051	209	280	255	195	173	237	281	418	201	6	✓	-
11	TV1051	209	280	255	195	173	237	281	418	201	6		

a Infections with day 0 versus recurrence differences are highlighted in bold.

b Microsatellite-based multi-locus genotype (MLG).

## DISCUSSION

Over the past 2 decades, several studies have investigated the involvement of *pvcrt-o* in conferring CQ resistance in P. vivax, but the findings remain contentious (8, 10–14, 18). A recent study using a genetic cross between CQS and moderate (surviving pediatric CQ doses) CQR parasite subpopulations of the NIH-1993-S × R strain linked *pvcrt-o* transcription to CQ resistance and 2 candidate markers in the gene region (MS334 and In9*pvcrt*) (12). Our study provides the first investigation of the MS334 and In9*pvcrt* variants using samples from a CQ clinical efficacy trial of patients with P. vivax malaria conducted in Sabah, Malaysia (16). During the period that the clinical isolates were collected, the study had well documented high-grade CQR. Our findings highlight that neither MS334 nor In9*pvcrt* reliably predict the clinical outcome following CQ treatment. Multi-locus genotypes (MLGs) derived from neutral microsatellite loci revealed a large clonal cluster that comprised infections from both the CQS and CQR patient groups. Potential explanations for the observed patterns at the neutral MLGs, MS334, and In9pvcrt loci are discussed.

The high proportion of Malaysian P. vivax infections with a single MLG type (MLG6) is indicative of unstable transmission, and potentially reflect clonal expansion, as previously described in this population (17, 19). At the time of the clinical trial, Malaysia was in the P. vivax pre-elimination stage, with intense P. vivax population bottlenecking (17). CQ was the first-line treatment throughout the study period and in view of its very poor efficacy, in 2016 national antimalarial policy was changed to recommend artemisinin-based combination therapies for the treatment of P. vivax.

The observation of equally high proportions of both CQS and CQR infections with the predominant MLG6 genetic backbone raises important questions concerning the genetic basis of CQ resistance in P. vivax in this endemic setting. It is plausible that factors such as patient immunity, variation in drug metabolism, or delayed recurrence (after 28 days) may have confounded the clinical response and thus the parasite phenotype. Indeed, the low prevalence and unstable transmission of P. vivax may have impacted on host immunity in this endemic setting. However, all patients treated with CQ in the clinical trial had observed administration with correct dosage based on body weight and confirmed therapeutic plasma CQ concentrations at day 7 (16). At the time of recurrence, 9 of 20 patients assessed in the original study had plasma CQ + DCQ concentrations more than 15 ng/mL (range, 19.7 to to 120.5), demonstrating parasite growth in the presence of adequate drug concentrations (16). By definition, these parasites were CQR. Furthermore, the risk of recurrence by Day 28 exceeded 60%, far greater than that expected under normal variation in a clinical trial (3). It is also noteworthy that in the same trial, there were no P. vivax recurrences by Day 42 in the artesunate-mefloquine arm (16). Another possible explanation for the CQ efficacy patterns at MLG6 is that the mechanism of CQ resistance in P. vivax entails within-host mutational selection that is undetectable in the isolate collected before treatment. A single cell sequencing study of 11 paired (Day 0 and recurrent time point) P. vivax isolates from Thailand found evidence for within-host blood-stage mutation and selection at a repertoire of genes, including the ApiAP2 family of transcription factors that have been shown to be targets of selection in endemic populations including Malaysia (17, 20–23). The detection of the MS334 and In9*pvcrt* loci using a genetic linkage approach refutes a non-heritable mechanism. Nonetheless, further studies are warranted to evaluate mutational changes at genes such as the ApiAP2 family between pretreatment and recurrent parasites.

A third explanation for the CQ efficacy patterns observed at MLG6 is that the microsatellites that were used to describe the parasite genetic backbone in the Malaysian cohort are transmitted independently from an underlying parasite genetic determinant(s) of CQR. In this scenario, the MLGs would not be expected to be associated with CQ treatment outcome (as we observed in our study), but the determinant(s) should be. There was a modest degree of independence between the MLG loci and the MS334 locus, but to a lesser degree with the In9*pvcrt* locus. A direct assessment of *pvcrt-o* expression may provide further insight.

The genetic cross conducted by Sá and colleagues (2019) identified variable numbers of TGAAGH motifs at MS334 and In9*pvcrt* as markers of *in vivo* CQ efficacy (in an *Aotus* host) in CQS and CQR progeny, and that CQR progeny had increased expression of *pvcrt*-o (12). However, it is unclear whether either of these indels has a causative role in determining the response to CQ through increased *pvcrt*-o expression or other mechanisms. Interestingly, a study of gene expression across life cycle stages in P. vivax isolates from Cambodia showed that *pvcrt*-o has multiple potential protein isoforms due to the retention of intron 9 that predicted an early stop codon (24). Another study, conducted on clinical isolates from Thailand and Indonesia, identified an AAG trinucleotide insertion in the *pvcrt-o* exon 1 encoding an extra lysine, associated with reduced CQ IC_50_s, although the insertion was observed predominantly in Thai isolates (76%) rather than Indonesian isolates (2.2%); the study was potentially confounded by population structure effects (7). TGAAGH motifs have been shown to regulate gene expression in *Arabidopsis*, but there is currently no knowledge regarding their role in *Plasmodium* (25, 26). In Malaysia, we observed a wide range of variable numbers in TGAAGH motifs in both MS334 and In9*pvcrt* among CQS and CQR isolates, with no evidence of a correlation between motif length and clinical outcome following treatment with CQ. For example, the Malaysian CQR isolate TV005 possessed the shortest number of TGAAGH motif lengths in both gene regions, while 1 CQR isolate collected at recurrence, MK2079, possessed the longest motif lengths in both gene regions. Understanding the structural impact of these repeat variations in such noncoding regions of a highly conserved gene, such as *pvcrt-o*, may help to explain parasite mechanisms of survival to lower levels of drug independence of amino acid changes in the PvCRT protein.

Although increased *pvcrt-o* expression has been associated with CQ resistance in several studies (10–12), this pattern has not been replicated in clinical data sets of populations with high-grade CQ resistance. Study of clinical P. vivax isolates from Papua, Indonesia, an area of high-grade CQ resistance, found no evidence of association between *pvcrt-o* expression and *ex vivo* CQ susceptibility, suggesting this gene is differentially expressed in blood stages, a problem for bulk RNA analysis previously reported (14). Furthermore, although a population genomic study using phenotype-free methods to detect selective sweeps identified a weak signal of selection in the vicinity of *pvcrt-o* in an Ethiopian population, this signal was not identified in similar studies conducted in either Malaysia or Papua, Indonesia, where high-grade CQ resistance is present (17, 21, 27). It is possible that different CQ resistance mechanisms are involved in different P. vivax populations. Alternatively, as discussed earlier, the CQ resistance mechanism may not be heritable, in which case studies applying population genomic approaches to detect sweeps or association methods using pretreatment clinical isolates may fail to identify a signal.

In the current study, we analyzed paired isolates collected at baseline and again on recurrence to explore any changes in the TGAAGH motif lengths after drug exposure. Although the sample size was limited, there was no evidence of an association between MS334 or In9*pvcrt* motif length and CQ resistance. Six paired isolates had different MS334 motif lengths pre and post treatment, however the direction of change (i.e., increase versus decrease in motif length) was inconsistent. Microsatellite-based MLGs confirmed that the post treatment isolates were the same strains as those present at pretreatment, except for 2 pairs, MV019/MV1019 and TK026/TK1026; in these pairs, the isolate at baseline was monoclonal, but the isolates at recurrence comprised at least 2 strains. It’s plausible that a polyclonal infection was present at both baseline and recurrence, but a minor CQR strain was only detected at recurrence following preferential growth under selective drug pressure.

Our study has several limitations. The extensive sequence complexity in the MS334 and In9*pvcrt* regions, and the high diversity of variants observed in the Malaysian population-imposed challenges in sequence alignment and variant calling. To overcome these challenges, we confined our study to high-quality monoclonal infections and 2 independent investigators, both blinded to the sample’s CQ sensitivity status and analyzed the molecular assemblies. As detailed above, we were unable to rule out the potential impact of host or other parasitological factors that might impact CQ treatment outcome in the Malaysian study, but we expect this to be minimal. It will be informative to assess the MS334 and In9*pvcrt* markers in other studies using other CQ sensitivity phenotypes, such as *ex vivo* drug susceptibility assays, which are less vulnerable to host confounding factors (28). *Ex vivo* phenotyping using methods, such as the schizont maturation assay, will need to address challenges, including defining a therapeutically relevant IC_50_ to define CQR, and the need to adjust for factors such as parasite density, developmental stage, and assay duration, which can impact the accuracy of phenotyping (29–31). Culturable heterologous model systems in closely related *Plasmodium* spp. such as P. knowlesi or P. cynomolgi present another approach to evaluate candidate markers in laboratories with expertise in these methods (28, 32). Our study was also limited by small sample size, although, despite the near equal proportions of the 2 treatment outcome groups, there was no trend in our findings.

In summary, our study revealed complexity in the relationship between parasite genetics and CQ resistance in a pre-elimination Malaysian P. vivax population with evidence of clonal expansion: in this endemic setting, we found that the MS334 and In9*pvcrt* markers were not reliable predictors of clinical outcomes following CQ treatment of patients with P. vivax. Further evaluation of these and other candidate markers in different endemic settings, and studies exploring the mechanism of P. vivax CQ resistance and the identification of molecular markers using candidate-free genome-wide approaches are warranted.

## MATERIALS AND METHODS

### Sample collection and selection.

Clinical isolates of P. vivax were collected from a comparative efficacy study of patients with uncomplicated *vivax* malaria conducted in Sabah, Malaysia, between 2012 and 2014 (Clinical Trials Registration number NCT01708876) (16). Briefly, consenting patients aged ≥1 year and weighing > 10kg, presenting with acute, uncomplicated *vivax* malaria to the study hospitals in Kudat, Kota Marudu, and Pitas were recruited into the prospective study. Patients were randomly assigned to either CQ (median total dose of 27.7 mg/kg [range, 25.0 to 33.0]) or artesunate-mefloquine (AS-MQ) (median 11.0 mg/kg [range, 9.8 to 12.0] AS and 27.6 mg/kg [range, 24.6 to 30.0] MQ), and followed for 42 days. Administration of primaquine for radical cure was delayed until 28 days of follow up. All treatment doses of chloroquine were supervised. The primary outcome was the cumulative risk of P. vivax parasitemia by day 28. Blood samples were collected at enrollment and on the day of recurrence for molecular analyses. Monoclonal P. vivax isolates collected at enrollment into the CQ treatment were selected for further molecular analysis in the *pvcrt-o* study. Isolates were categorized as CQS if the patient exhibited adequate parasite clearance and no recurrent parasitemia within 42 days. If patients failed to clear peripheral parasitemia on blood smear or presented with recurrent P. vivax parasitemia within 42 days after CQ treatment in the presence of a minimum effective plasma concentration of chloroquine and desethylchloroquine (CQ + DCQ) of ≥ 15ng/mL at Day 7 (corresponding to a whole blood concentration of 100 ng/mL) (33), they were defined as CQR (16, 33). All patient samples and corresponding clinical data were assigned unique study code identifiers (not identifiable to anyone outside the research group) to protect the identity of the subjects.

### DNA extraction, speciation, and genotyping.

DNA was extracted from 200 μL whole blood using the QIAamp DNA Blood extraction kit according to the manufacturer’s procedures (Qiagen). *Plasmodium* spp. was confirmed using the multiplex PCR method described by Padley et al. (34). P. vivax genotyping data was derived from published data stored in the open access vivaxGEN-MS platform (https://vivaxgen.menzies.edu.au) using batch code MYPV-MG and applying the default parameters for genotype calling (35). Briefly, genotyping was performed using capillary electrophoresis at 9 short tandem repeat (STR) markers to determine the multiplicity of infection (MOI) as previously described (19).

### *pvcrt-o* gene fragment amplification and sanger sequencing.

The primer sequences and PCR cycling conditions are summarized in Table 5. The PCR products were run on 2% agarose gel, stained with SYBR Safe DNA Gel Stain (Invitrogen) and visualized using the Molecular Imager Gel Doc XR system (Bio-Rad). PCR amplification of In9*pvcrt* yielded multiple bands and henceforth gel extraction was required prior to sequencing, as conducted on previous experiments. Amplicons were sent to Macrogen Inc (Seoul, South Korea), where PCR product purification by gel extraction was conducted prior to sequencing using the dideoxy termination method and the respective PCR primers.

**TABLE 5:** Primer sequences and PCR cycling conditions^*a*^

Primer pair	Primer sequence (5′ – 3′)	PCR cycling condition
MS334_F	GAAATGTAGATTTAAGTGCA	94°C for 2 min, 40 cycles of (94°C for 30 sec, 48°C for 45 sec, 72°C for 45 sec), 72°C for 5 min
MS334_R	TGTCACTTTGTCAAATAACA	
Intron9_F	GGTTGGAATGCCAGATG	94°C for 2 min, 40 cycles of (94°C for 30 sec, 55°C for 45 sec, 72°C for 45 sec), 72°C for 5 min
Intron 9_R	TCGCAAATGTTGAAAAAGGA	

a PCR, polymerase chain reaction.

### Sequence analysis.

Trimming low-quality ends of the sequences was performed using Mega-X software (Version 10.2.5). Post-trimming, manual assembly of contigs, and comparison with the gene regions of the CQS reference Sal-1 strain were performed using BioEdit software (Version 7.2.5). Alignments were undertaken with the bioinformatician blinded to the CQ treatment outcomes of the patients. To ensure consistency in assemblies between the genetic cross study (12) and the current study, the NIH-1993-S and NIH-1993-R sequences described in the former study were analyzed alongside the Malaysian assemblies.

### Statistical analysis.

All statistical tests were conducted using in-built functions of R (Version 2022.12.0). Proportions were assessed using the Chi-squared test with Yates’ correction and Wilcoxon test was used for non-parametric comparisons. A significance threshold of *P < *0.05 was used for all statistical comparisons.

### Ethics.

All samples were collected with written informed consent from patient or legal guardian for individuals under 18 years of age. Ethical approval for the patient sampling and parasite molecular analysis was provided by the Human Research Ethics Committee of the Northern Territory Department of Health and Families (HREC-2010-1431, HREC-2012-1815 and HREC-2010-1396) and the National Medical Research Ethics Committee, Ministry of Health, Malaysia (NMMR-10-754-6684, NMRR-12-511-12579).
