# Supplementary figures and images for: No Association between the Plasmodium vivax crt-o MS334 or In9pvcrt Polymorphisms and Chloroquine Failure in a Pre-Elimination Clinical Cohort from Malaysia with a Large Clonal Expansion
Source: Antimicrob Agents Chemother. 2023 Jun 14;67(7):e01610-22. doi: 10.1128/aac.01610-22 (PMC10353443; doi:10.1128/aac.01610-22)

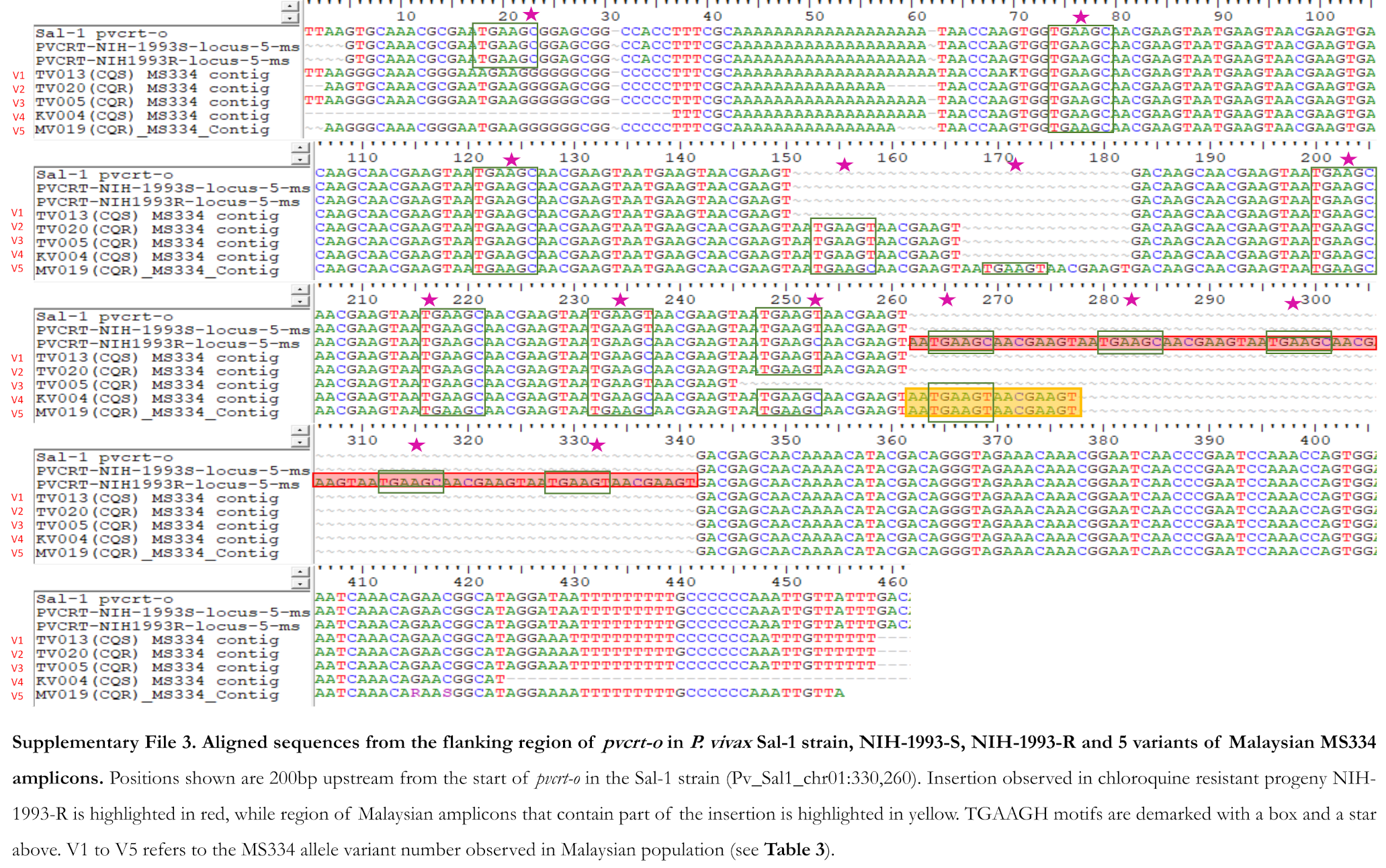

Supplement: Supplemental file 3 — Supplemental material. Download aac.01610-22-s0003.tif, TIF file, 2.8 MB [file aac.01610-22-s0003.tif]

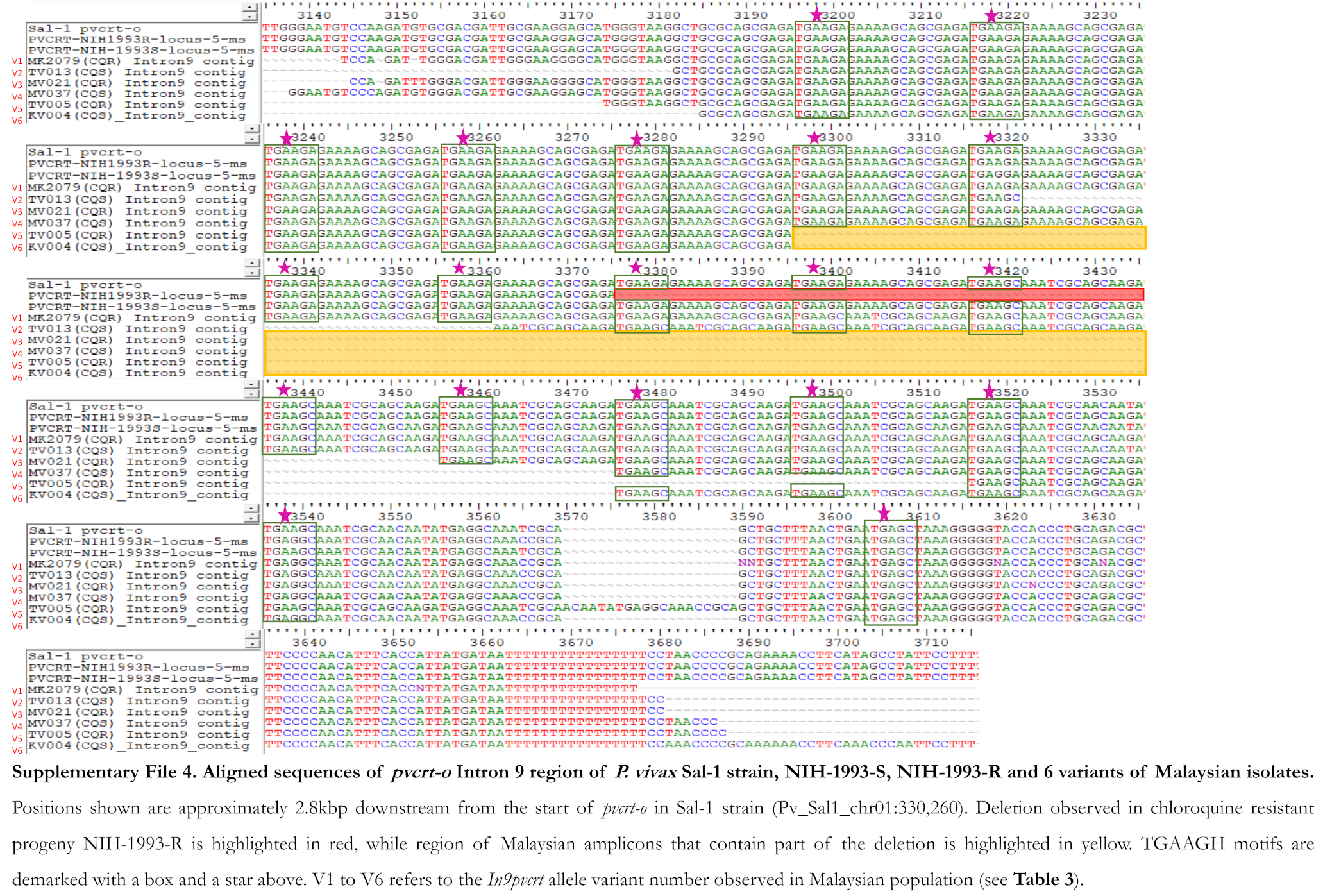

Supplement: Supplemental file 4 — Supplemental material. Download aac.01610-22-s0004.tif, TIF file, 3.2 MB [file aac.01610-22-s0004.tif]
